# Steam-Exploded Pruning Waste as Peat Substitute: Physiochemical Properties, Phytotoxicity and Their Implications for Plant Cultivation

**DOI:** 10.3390/ijerph19095328

**Published:** 2022-04-27

**Authors:** Rui Yang, Xuejiao Chen, Dongdong Zhang, Hong Wang, Wanlai Zhou, Wei Lin, Zhiyong Qi

**Affiliations:** 1 Institute of Urban Agriculture, Chinese Academy of Agricultural Sciences, Chengdu 610213, China; yangrui01@caas.cn (R.Y.); dongdongzhang.ac@outlook.com (D.Z.); wanghong07@caas.cn (H.W.); zhouwanlai@caas.cn (W.Z.); linwei01@caas.cn (W.L.); 2 School of Food and Biotechnology, Xihua University, Chengdu 610039, China; xjchen@cau.edu.cn

**Keywords:** steam explosion, pruning waste, growing substrate, temperature, retention time, phytotoxicity, biomass, phenol, flavonoid, alkaloid

## Abstract

Peat is a nonrenewable resource that we are using at alarming rates. Development of peat alternative from pruning waste (PW) could be a cost- and environment-friendly way of disposal. Steam explosion (SE) is a commonly used pretreatment of lignocellulosic biomass, but its impact on the properties of PW as a growing substrate is largely unknown. To address this issue, PW was treated using five SE temperatures (160, 175, 190, 205 and 220 °C) and three retention times (1, 3 and 5 min) and evaluated for key traits of growing substrate. Results indicate that bulk density, total porosity, EC, total carbon, and concentration of phytotoxins including phenol, flavonoid, and alkaloid significantly increased or tended to increase with increasing temperature and/or retention time. A reversed trend was observed for water-holding capacity, pH, content of hemicellulose and lignin, and germination index. Cation exchange capacity and total N showed minimal response to SE. Steam explosion had inconsistent impacts on acid soluble nutrients. Phytotoxicity was a major factor limiting the use of SE-treated PW as growing substrate. Higher pretreatment severity led to higher phytotoxicity but also facilitated subsequent phytotoxicity removal by torrefaction. Pruning waste treated by SE and torrefaction under certain conditions may be used as peat substitute for up to 40% (v/v).

## 1. Introduction

The booming soilless culture industry globally creates a huge demand for soilless growing substrate [1]. Annual requirement for professional growing substrate in China is estimated to be 86 million m^3^, but only <12.5% is fulfilled [2]. Peat is the most widely used organic growing substrate. However, the environmental and economic concerns associated with peat has urged researchers and growers to find more cost-effective and environment-friendly peat alternatives [3].

On the other hand, rapid urbanization over the past few decades leads to a remarkable increase in urban green space as well as the production of pruning waste (PW) such as tree wood and barks, grass clippings, and leafy residues. According to the National Bureau of Statistics of China, urban green space has reached 3.3 million hectares in 2020 and over 350 million tons of PW was generated [4]. Pruning waste has thus become a major constituent (e.g., 22–30%) of municipal solid waste by weight [5]. Though PW has been utilized for compost and biofuel production, most PW is still buried in landfill and incinerated posing a great challenge to the management of urban environment [4,6]. Development of growing substrate from PW may help solve the disposal problem and partially replace peat.

Composting, particularly co-composting with other organic wastes such as animal manure, sewage sludge and food-processing waste, is the most widely adopted pretreatment of PW before using it as growing substrate [7,8,9]. However, varied composition, high bulk density, excess salt and relatively long preparation time have been major factors limiting green compost as growing substrate [10]. According to the German Compost Quality Assurance Organization, compost cannot be used as a stand-alone growing substrate and should not exceed 40% (v/v) in potting soil [11]. Solid products from thermal treatments (e.g., pyrolysis and hydrothermal carbonization) of PW including biochar and hydrochar have also been evaluated as a growing substrate [3,12,13]. However, biochar and hydrochar vary in properties dependent on feedstocks and are relatively high in production cost [14]. The presence of phytotoxins and high N immobilization in hydrochar also restricts plant growth [13]. 

Steam explosion (SE) is one of the most commonly used pretreatments of biomass due to its lower cost and energy demand and less environmental impact compared to other treatments [15]. For this process, biomass is treated by saturated steam at high temperatures (140–220 °C) for several minutes followed by a sudden release of pressure. As a result, the chemical bonds break down and macromolecules partially depolymerize, which greatly reduces the recalcitrance of biomass [16]. Therefore, SE is widely used in bioethanol production and biorefinery processes [17]. To date, few studies have evaluated SE as a pretreatment of lignocellulosic biomass for growing substrate. Chen et al. (2019) found that SE could decrease average pore size and thus increase specific surface area of rice and oil-seed rape straw [16]. Such an increase may result in improved water and nutrient retention, which is a favorable trait as a growing substrate. However, SE pretreatment might not be suitable for all feedstocks due to their highly heterogeneous nature. Strong interest in understanding the suitability of SE-treated PW as a growing substrate has thus prompted this research to thoroughly determine the impacts of different SE conditions on physiochemical properties and the phytotoxicity of PW.

## 2. Materials and Methods

### 2.1. Collection and Treatment of PW

Pruning waste was collected from the campus of the Chengdu Academy of Agriculture and Forestry Sciences, Chengdu, Sichuan, China (30.71° N, 103.86° E), and were mainly composed of woody materials (e.g., stems and barks) from local species including approximately 18% *Jacaranda mimosifolia*, 20% *Osmanthus fragrans*, 8% *Elaeocarpus sylvestris*, 11% *Morus alba* and 43% *Cinnamomum camphora* by weight. The feedstocks were air dried, ground to pass a 3-cm sieve, and thoroughly mixed.

Feedstocks were steam exploded at temperatures ranging between 160 and 220 °C with an increment of 15 °C using a QB-300 instant catapult steam explosion (ICSE) apparatus (Tsing-Gentle Eco-Technology Co., Ltd., Suzhou, China; Figure S1). Retention time was 1, 3, and 5 min for each temperature, resulting in a total of 15 temperature/retention time combinations and pretreatment severity (LogR_0_) [18] ranging between 1.77 and 4.23 (Table S1). An untreated control treatment was also included.

Untreated PW and PW that were steam-exploded with low (160 °C + 3 min), medium (190 °C + 3 min), and high (220 °C + 5 min) pretreatment severity were selected to receive torrefaction treatment following procedures described in our previous works [19,20] for phytotoxicity removal analysis.

Untreated PW and SE-treated PW were then dried in an air-forced oven at 75 °C for 48 h and ground to pass a 2-mm sieve for subsequent analysis in triplicates.

### 2.2. Analysis of Physiochemical Properties

Bulk density (BD), total porosity (TP), air-filled porosity (AFP), and water-holding capacity (WHC) were measured using the ring knife method [21]. Briefly, weight of a 60 cm^3^ cutting ring was first recorded (w1). The cutting ring was then filled with oven-dried sample (without compression) and weighed (w2). After the sample was saturated by soaking in water for 24 h, the cutting ring was weighed (w3). The cutting ring was then placed on a dry sand layer and water was allowed to drain by gravity for 3 h before it was weighed again (w4). Bulk density (BD), total porosity (TP), water-holding capacity (WHC), and air-filled porosity (AP) were calculated using the following equations:

$${\mathrm{Bulk}\;\mathrm{density}\;(g\;\mathrm{cm}}^{-3})=\frac{w2\;-\;w1}{60}$$

$$\mathrm{Total}\;\mathrm{porosity}\;(\%\;v/v)=\frac{w3\;-\;w2}{60}\;\times \;100$$

$$\mathrm{Water}\text{-}\mathrm{holding}\;\mathrm{capacity}\;(\%v/v)=\frac{w4\;-\;w2}{60}\;\times \;100$$

$$\mathrm{Air}\text{-}\mathrm{filled}\;\mathrm{porosity}\;(\%\;v/v)=\frac{w3\;-\;w4}{60}\;\times \;100$$

pH and electrical conductivity (EC) were analyzed with a sample/water ratio of 1:10 (w/v) using a Mettler-Toledo FE28 pH meter and a Mettler-Toledo FE38 conductivity meter (Mettler-Toledo Instruments Co., Ltd., Shanghai, China). Cation exchange capacity (CEC) was determined following the method described by Sumner and Miller (1996) [22]. Briefly, 5 g sample was saturated with 60 mL 1 M NH_4_OAc solution and washed using 60 mL ethanol to remove excessive NH_4_^+^. Then the remaining sample was washed with 60 mL 1M KCl solution. Concentration of NH_4_^+^ in the leachate was analyzed using the Kjeldahl method for calculation of CEC. Proximate analysis was performed following the ASTM-D5142-04 method [23]. Chemical composition including the content of hemicellulose, cellulose, and lignin was analyzed following the method proposed by the National Renewable Energy Laboratory [24]. Total carbon and nitrogen were determined using a vario EL cube elemental analyzer (Elementar Analysensysteme GmbH, Langenselbold, Germany). Acid soluble nutrients including K, Na, Ca, Mg, and P were extracted using Mehlich 1 soil test [25] with a modified sample/extractant ratio of 1:6 (w/v) and were analyzed using an Agilent 7700e inductively coupled plasma mass spectrometry (Agilent Technologies, Inc., Santa Clara, CA, USA).

### 2.3. Analysis of Phytotoxicity

Phytotoxicity was evaluated as germination index (GI) [26]. Briefly, a germination paper was placed in a petri dish and 10 mL of aqueous extract (1/10, w/v) from the sample was added. Deionized water was used as the control. Ten seeds of bok choy (*Brassica rapa* subsp. *Chinensis* cv. Jingguan) were placed on the germination paper and incubated in the dark at 25 °C for 48 h in a germinator. The germination percentage and the root length were then determined. The GI was calculated as

$$\mathrm{GI}=\frac{G_{\mathrm{sample}}}{G_{\mathrm{control}}}\;\times \;\frac{L_{\mathrm{sample}}}{L_{\mathrm{control}}}$$

where G_sample_ and G_control_ were the percentage of germinated seeds for the sample and the control, and L_sample_ and L_control_ were the mean root length for the sample and the control.

Potential phytotoxic chemicals were also determined. Concentration of total phenol and total flavonoid were analyzed following colorimetric methods reported by Wang et al. (2020) [27] using commercial testing kits (SolarBio Life Sciences, Beijing, China). Concentration of total alkaloid was determined using colorimetric method with Reinecke salt (NH_4_[Cr(NH_3_)_2_(SCN)_4_]). Briefly, 1 g sample was soaked in 10 mL 0.005 M HCl for 12 h, ultrasonicated for 25 min, and centrifuged at 10,000× *g* for 5 min. The supernatant was passed through D101 macroporous adsorption resin. The leachate was collected, homogenized with 5 mL 2% Reinecke salt solution, and incubated in an ice bath for 30 min. The precipitant was collected after filtration, washed with deionized water, freeze-dried, and re-dissolved in 5 mL acetone for absorbance determination at 525 nm. Ligustrazine hydrochloride was used as the standard reference.

### 2.4. Plant Cultivation

Seeds of bok choy (*Brassica rapa* subsp. *Chinensis* cv. Jinguan) were sown into 72-cell plug trays filled with commercial peat (Pindstrup Horticulture Co., Ltd., Shanghai, China) on 9 November 2021. Key characteristics of the commercial peat include particle size 0–6 mm, pH 6.5, EC 0.74 mS cm^−1^, CEC 35.5 cmol_c_ kg^−1^, bulk density 0.19 g cm^−3^, total porosity 86.3% (v/v), air-filled porosity 20.7% (v/v), water-holding capacity 65.6% (v/v), and C/N ratio 48.2.

Seeds were germinated in a growth chamber with day/night temperatures of 24/22 °C and relative humidity (RH) of 60% under LED lights with a photosynthetic photon flux density of 150 µmol m^−2^ s^−1^ and a photoperiod of 16 h. Upon unfolding of the first pair of true leaf (23 November 2021), uniform seedlings were transplanted to plastic pots that had drainage holes at the bottom and were filled with 250 cm^3^ growing substrate. The commercial peat was mixed with vermiculite and perlite at a ratio of 3:1:1 by volume as the control treatment for plant cultivation. Untreated PW and SE-treated PW was used to replace the commercial peat at 5, 10, 20 and 40%. Each treatment was analyzed in triplicates. 

Seedlings were cultivated in the same growth chamber. Each pot was irrigated weekly with 60 mL modified Hoagland nutrient solution containing 4 mM KNO_3_, 0.8 mM KH_2_PO_4_, 0.3 mM K_2_HPO_4_, 1.5 mM MgSO_4_, 3 mM Ca(NO3)_2_, 0.08 mM Fe-Na EDTA, 60 µM H_3_BO_3_, 3 µM ZnSO_4_, 20 µM MnSO_4_, 0.4 µM CuSO_4_ and 0.03 µM (NH_4_)_6_Mo_7_O_24_. Seedlings were harvested on 17 December, 2021. Chlorophyll was measured on the 2nd pair of fully-expanded leaf from the top immediately prior to harvest using a SPAD meter (Zhejiang Top Cloud-Agri Technology Co., Ltd., Hangzhou, China). Shoot fresh weight was first measured. After drying in an air-forced oven at 75 °C for 48 h, shoot dry weight was measured.

### 2.5. Statistical Analysis

Data were analyzed using mixed model methodology. For steam explosion, fixed effects included temperature and retention time. For plant cultivation, fixed effects included SE treatment (i.e., temperature/retention time combinations), torrefaction, and percentage of peat substitution. Replicate was treated as the random effect. Statistical analysis was implemented using SAS 9.4 software (SAS Institute, Cary, NC, USA) for comparing the differences among fixed effects and their interactions. *p* values were adjusted for multiple comparisons using Tukey’s Honestly Significant Difference test.

## 3. Results

### 3.1. Impact of SE on Physical Properties of PW

Bulk density, TP, and WHC for the untreated PW were 0.21 g cm^−3^, 64.8% (v/v), and 59.9% (v/v), respectively, and were significantly improved following SE. These three physical traits were significantly affected by main effects of SE temperature and retention time (*p* < 0.001) but not their interactions (*p* > 0.05).

Bulk density gradually increased with increasing temperature or retention time with or without statistical significance (Table 1). The greatest TP and WHC occurred at 175 °C and gradually decreased as temperature further increased, but such decrease did not reach statistical significance for WHC (Table 1). Steam explosion for 5 min resulted in notably greater TP and WHC than that for 3 min, but differences between 1 and 3 min or between 1 and 5 min were not significant (Table 1). Air-filled porosity did not respond to steam explosion (*p* = 0.225). Mean AFP across all SE treatments was 6.64% compared to 4.90% for the untreated control (Table 1).

### 3.2. Impact of SE on Chemical Properties of PW

pH, EC, CEC, hemicellulose, cellulose, lignin, total C, C/N ratio, and acid soluble Na and Ca were significantly affected by temperature×retention time interaction (*p* < 0.001). pH of the untreated PW was 6.58 and significantly decreased to as low as 4.77 following SE (Table 2). At a given temperature, pH decreased as retention time increased, and vice versa. EC of the untreated PW was 0.85 mS cm^−1^. All SE treatments except for the one with lowest pretreatment severity (i.e., 160 °C + 1 min) significantly increased EC compared to the untreated control (Table 2). In contrary to pH, EC tended to increase with increasing temperature and retention time.

Among all temperature/retention time combinations, only the 160 °C + 3 min treatment significantly increased CEC compared to the untreated control (6.2 cmol_c_ kg^−1^). Differences among retention times at each temperature were not significant and vice versa (Table 2). 

Hemicellulose content in untreated PW was 222 mg g^−1^ and significantly reduced following SE excluding the two treatments at 160 °C for 1 and 3 min (Table 2). At each retention time, hemicellulose content gradually decreased with increasing temperature. At 160 and 175 °C, hemicellulose content between 1 and 3 min was similar and further decreased by ~20% when retention time increased to 5 min. At higher temperatures, hemicellulose content gradually decreased with increasing retention time, but differences between 3 and 5 min at 220 °C were not significant (Table 2). 

Cellulose in untreated PW was 387 mg g^−1^ and did not notably change following SE (Table 2). Steam explosion treatments with a LogR_0_ > 2.65 significantly decreased lignin by 10.3–27.1% compared to the untreated control (321 mg g^−1^; Table 2). Impacts of temperature and retention time on cellulose and lignin content in PW highly varied and did not follow specific patterns (Table 2). 

Total C in the untreated control was 44.6% and was significantly increased by SE treatments with a LogR_0_ ≥ 3.09 (Table 2). At 205 and 220 °C, total C tended to increase with increasing retention time. Total N was 0.28% in the untreated control and showed no response to SE treatments (*p* = 0.214). C/N ratio was 159 in the untreated control and significantly reduced following SE, but differences in C/N ratio among temperatures or retention times were mostly not significant (Table 2). 

Evaluated nutrients showed highly varied response to SE treatments (Table 2). The 160 °C + 5 min treatment significantly increased acid soluble Na compared to the untreated control (56.6 mg kg^−1^), whereas none of the SE treatments significantly altered acid soluble Ca relative to the untreated control (483 mg kg^−1^). Concentration of both acid soluble Na and Ca tended to increase with increasing retention time at 160 and 220 °C, but an opposite trend was observed at 175 and 190 °C. Acid soluble P significantly increased following SE but was not significantly different among temperature/retention time combinations (Table 2). Acid soluble K and Mg did not respond to SE (*p* > 0.05). Mean concentration of acid soluble K and Mg across all SE treatments was 1.38 and 0.70 g kg^−1^, comparing to 1.27 and 0.64 g kg^−1^ in untreated PW (data not shown). 

Steam explosion did not have significant impact on proximate analysis (*p* > 0.05). Mean content of VM, FC, and ash across all SE treatments was 80.7, 17.0, and 2.28%, respectively, compared to 80.3, 17.6, and 2.14% in the untreated control (data not shown).

### 3.3. Impact of SE and Torrefaction on Phytotoxicity of PW

**Figure 1:**
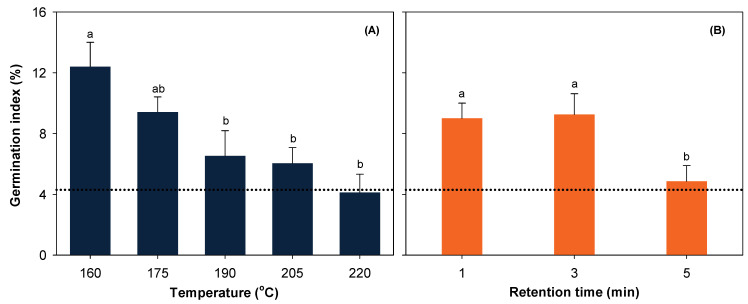
Germination index as affected by main effects of temperature (**A**) and retention time (**B**). Data represents mean ± SE. The dotted line represents untreated control. Means followed by different lower-case letters indicate significant differences among temperatures or retention times at α = 0.05.

Germination index was 4.3% for untreated PW and was significantly affected by main effects of temperature and retention time (*p* < 0.001) rather than their interactions. Steam explosion at 160 and 175 °C (Figure 1A) or for 1 and 3 min (Figure 1B) notably improved GI relative to the untreated control. Higher temperatures or longer retention times resulted in GI that was not significantly different from the untreated PW.

**Figure 2:**
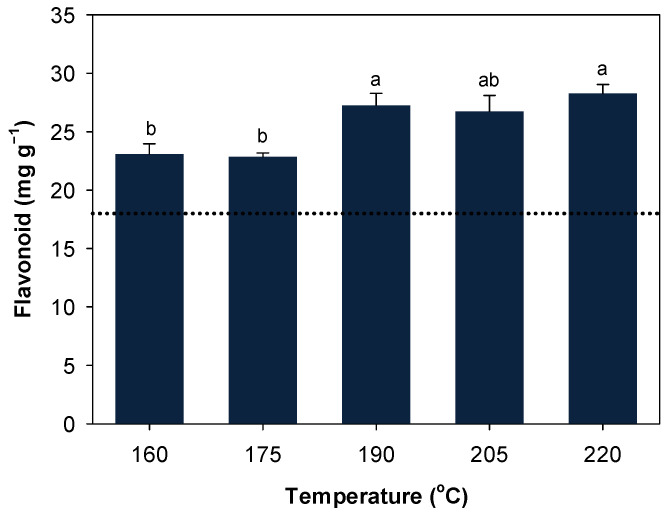
Concentration of total flavonoid as affected by main effect of temperature. Data represents mean ± SE. The dotted line represents untreated control. Means followed by different lower-case letters indicate significant differences at α = 0.05.

Concentration of total flavonoid was significantly affected by main effect of temperature (*p* < 0.001). Total flavonoid was similar between 160 and 175 °C and increased by >15% as temperature further increased (Figure 2). Differences among 190, 205, and 220 °C were not significant. 

Concentration of total phenol and total alkaloid was significantly affected by temperature × retention time interaction (Table 3). Steam explosion significantly increased total alkaloid compared to the untreated control (1.0 mg g^−1^). Total alkaloid gradually increased with increasing retention time at 160 and 175 °C. In contrast, SE for 5 min resulted in significantly decreased total alkaloid than that for 1 and 3 min at 205 and 220 °C (Table 3). Steam explosion treatments with a LogR_0_ < 3.0 did not significantly affect total phenol relative to the untreated control (10.2 mg g^−1^). At 190, 205, and 220 °C, total phenol significantly increased or tended to increase with increasing retention time, and vice versa (Table 3).

Germination index significantly improved following torrefaction (Table 4). For the untreated control, GI increased to 23.0% following torrefaction, but for SE-treated PW, torrefaction increased GI to 46.2–54.7%. Such results indicate that SE may facilitate phytotoxicity removal by torrefaction. Total alkaloid and flavonoid in evaluated SE treatments significantly decreased following torrefaction except that torrefaction increased total alkaloid by 23.3% in SE-treated PW at 190 °C + 3 min. Torrefaction also tended to decrease total phenol, but this reduction was only significant in SE-treated PW at 220 °C + 5 min (Table 4), indicating greater pretreatment severity of SE may facilitate removal of phenol by torrefaction. 

Torrefaction also affected certain physiochemical properties other than phytotoxicity. pH of torrefied PW with or without SE pretreatment was 6.42–6.50 (Table 4). Torrefaction reduced EC of PW and this decrease was significant for SE-treated PW with medium and high pretreatment severity. Increased pH and decreased EC made PW more suitable as growing substrate. Total C significantly increased following torrefaction, but total nitrogen was not significantly affected despite an increasing trend. Only C/N of the untreated control significantly dropped following torrefaction (Table 4).

### 3.4. Impact of SE-Treated PW on Plant Cultivation

**Figure 3:**
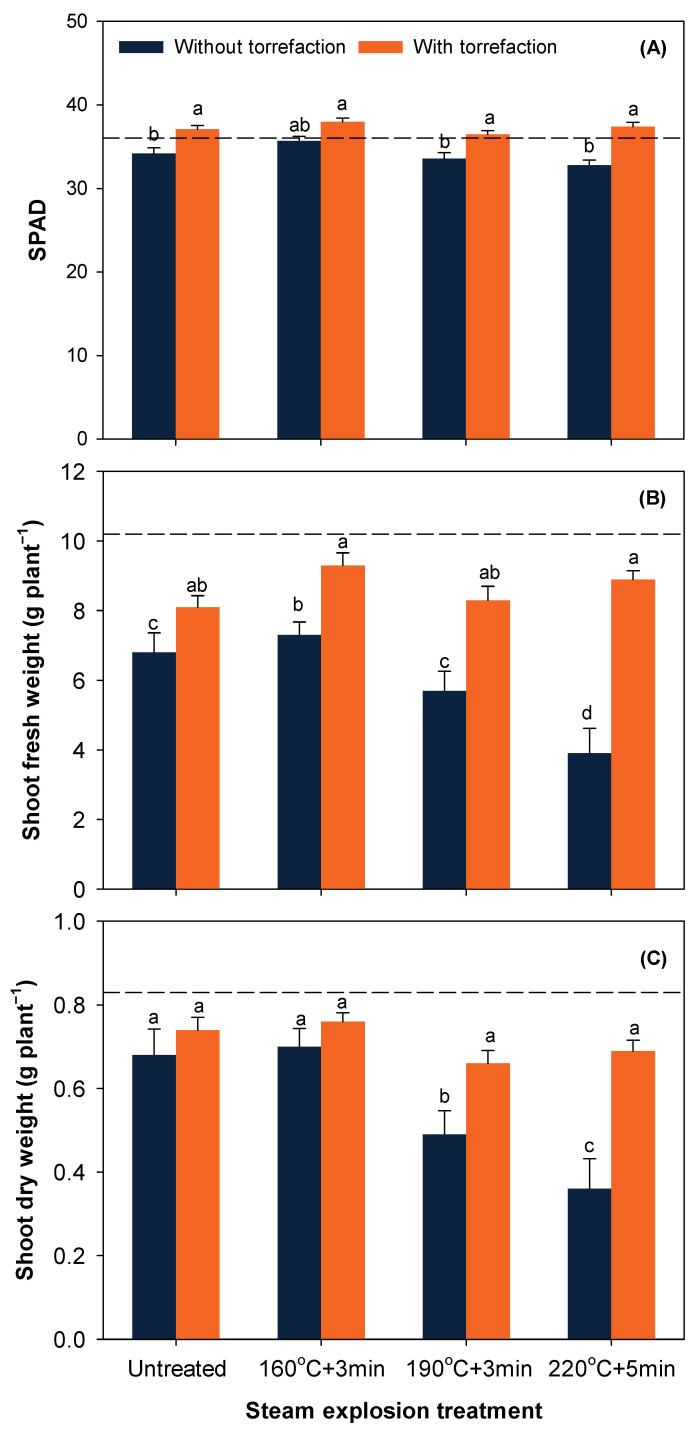
SPAD (**A**), shoot fresh weight (**B**), and shoot dry weight (**C**) of bok choy at harvest as affected by treatment × torrefaction interaction. The horizontal dash line represents bok choy grown in 100% commercial peat. Data represent mean ± SE. For each figure, means followed by different lower-case letters indicate significant difference at α = 0.05.

SPAD and shoot biomass was significantly affected by treatment×torrefaction (*p* < 0.001) and peat substitution × torrefaction interactions (*p* < 0.001). SPAD did not significantly differ among the four SE treatments with or without torrefaction (Figure 3A). Significantly increased SPAD in three of the four SE treatments following torrefaction suggested a better growing status. Shoot fresh and dry weight decreased with increasing pretreatment severity of SE, possibly due to increased phytotoxicity (Figure 3B,C). Torrefaction significantly increased shoot fresh weight of all four treatments (Figure 3B), but improvement in shoot dry weight was observed only for the two SE treatments with medium and high pretreatment severity (Figure 3C). Difference in shoot biomass among the four SE treatments diminished after torrefaction (Figure 3). 

**Figure 4:**
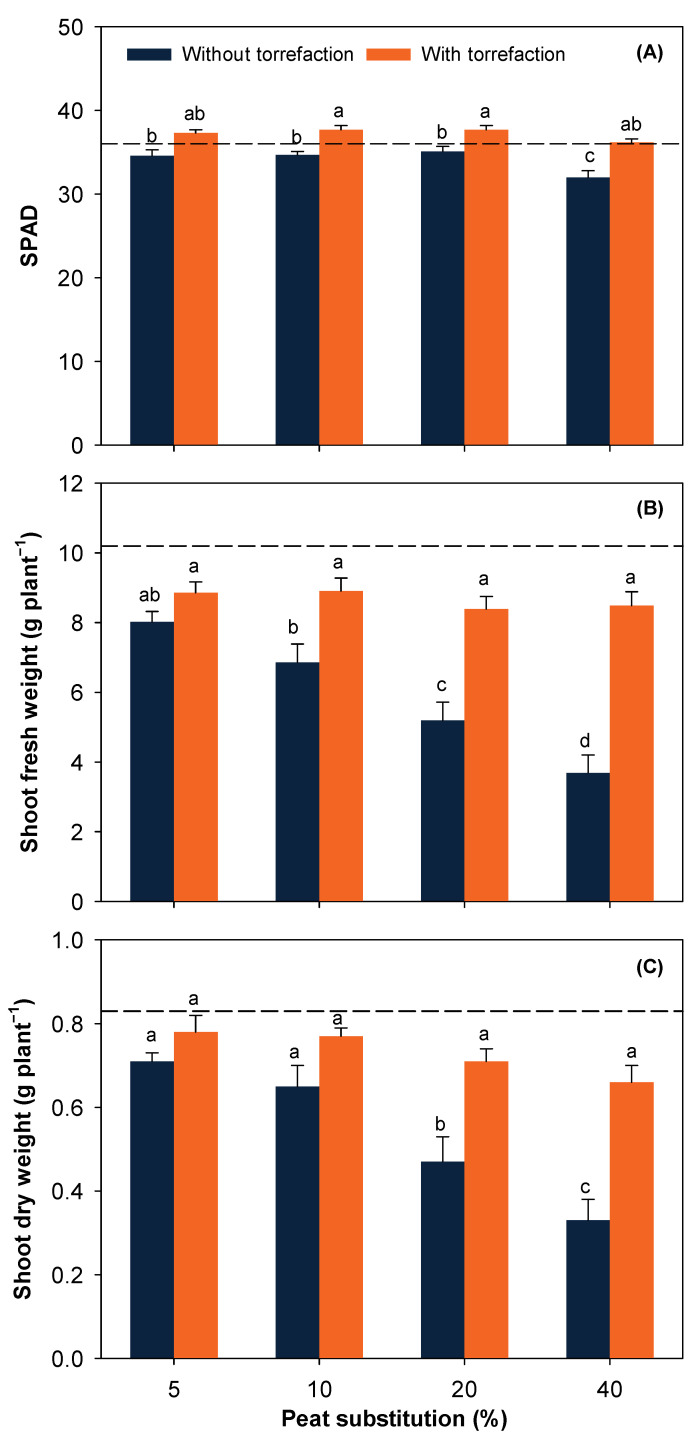
SPAD (**A**), shoot fresh weight (**B**), and shoot dry weight (**C**) of bok choy at harvest as affected by peat substitution×torrefaction interaction. The horizontal dash line represents bok choy grown in 100% commercial peat. Data represent mean ± SE. For each figure, means followed by different lower-case letters indicate significant difference at α = 0.05.

In the absence of torrefaction, SPAD significantly decreased at 40% peat substitution compared to lower substitution rates (Figure 4A). Torrefaction significantly increased SPAD except for 5% peat substitution. Shoot fresh and dry weight were not significantly different between 5 and 10% peat substitution and notably dropped as the substitution rate of SE-treated PW receiving no torrefaction further increased (Figure 4B,C). Torrefaction notably improved both shoot fresh and dry weight at higher peat substitution rates (i.e., 20 and 40%) rather than that at 5 and 10% peat substitution. Improvement in SPAD and shoot biomass indicates that torrefaction may reduce phytotoxicity and thus improve plant growth particularly for SE-treated PW with pretreatment severity.

## 4. Discussion

### 4.1. Physical Properties

Increased BD may be attributed to the reduced particle size resulting from a decreased diameter of fibers [28,29] and improved grindability resulting from weaken or destroyed plant cell wall structure [30,31] following SE. Scherzinger et al. (2020) found that the proportion of fine particles (i.e., 0.36–0.63 mm) in green waste increased following autoclave treatment, which is similar to steam explosion but has a weaker shearing force during pressure release [32]. Growing substrate with a bulk density of <0.3 g cm^−3^ is preferred by manufacturers [33]. The BD of SE-treated PW is below or close to this level and is thus not a factor limiting its subsequent use as growing substrate.

Growing substrate with a TP of ≥50% and a WHC of ≥40% is considered acceptable [33], and the TP and WHC of SE-treated PW meet this criterion. Air-filled porosity of <10% typically causes aeration limitation for plant growth, thus most commercial growing substrates have an AFP of 10–30% [34]. The low AFP of PW in this study may be explained by the fine particle size resulted from grinding and passing a 2-mm sieve following SE, as a negative correlation between particle size and AFP was previously reported in peat [35]. Since the particle size distribution of the growing substrate can be easily adjusted during processing, the low AFP of SE-treated PW can be corrected by increasing the proportion of larger particles.

### 4.2. Chemical Properties

Decreased pH following SE is likely due to the release of weak acids following SE, namely acetic and uronic acids during hydrolysis of hemicellulose [36] and formic and levulinic acids during degradation of furfural and 5-hydroxymethyl furfural [37]. Increased EC was expected as SE promotes hydrolysis of structural polysaccharides (e.g., hemicellulose and pectin) and extraction of water-soluble sugars. A positive correlation between solubilization and pretreatment severity of SE was previously reported [38].

Steam explosion had minimal impact on CEC of PW in this study. Though CEC is a critical factor determining availability and uptake of nutrients in addition to pH, Fields and Gruda (2021) argued that the impact of CEC on nutrient management in soilless growing substrate was not as important as in soil since the precise distribution of nutrient solutions in soilless culture makes the demand for a strong buffer capacity less critical [39].

Previous studies found that SE could efficiently decompose hemicellulose in a range of plant-based materials [16,40] in agreement with our work. In this study, cellulose content in PW showed minimal responses to SE treatments. Sui et al. (2019) [38] also found that SE (LogR_0_ 1.07–2.83) did not significantly change cellulose content in tea waste. However, Chen et al. (2019) [16] reported a 5–16% reduction in cellulose content in five crop straws following SE at 210 °C for 2 min (LogR_0_ = 3.54), whereas Semwal et al. (2019) [41] reported a 38% increase in cellulose content in rice straw following SE at 200 °C for 10 min (LogR_0_ = 3.94). Inconsistent results in the literature indicate that response of cellulose to SE varies contingent on the structural characteristics of the feedstocks and treatment conditions.

Steam explosion treatments with high pretreatment severity decreased lignin in PW. A 29% reduction in lignin was also reported in maize straw by Chen et al. (2019) [16]. Such decrease in lignin is probably because SE could effectively break the ether bond between hemicellulose and lignin [42]. With more hemicellulose losing under high pretreatment severity conditions, a more intensive lignin autohydrolysis may occur and result in the reduced lignin content [16]. In contrast, increased lignin following SE was also observed and was attributed to the formation of polymeric lignin-like materials (“pseudo-lignin”) under high pretreatment severity conditions [38,41].

Chemical composition has an important implication for biostability of organic growing substrate besides their impacts on physiochemical properties. Organic growing media may undergo microbial decomposition, which competes with plants for oxygen consumption and weakens the mechanic strength of growing substrate [34]. Cellulose is coated or sheathed by hemicellulose and acts as a blocking seal restricting the access of microbial enzymes such as cellulase and hemicellulose. High lignin content also contributes to low microbial degradability by forming a physical barrier through encapsulation with the cellulose–hemicellulose complex [43,44]. In this study, readily available substrate for microbial degradation such as hemicellulose decreased following SE, leading to an increased biostability, but meanwhile, decreased lignin under high pretreatment severity conditions contributed to decreased biostability to some extent. Apparent shrinkage or swelling of SE-treated PW and hypoxia was not observed in this study during plant cultivation, suggesting overall good short-term biostability of PW. Long-term biostability of SE-treated PW still needs further investigation. 

A C/N ratio of >35 may cause N immobilization, but potential N immobilization due to high C/N ratio in SE-treated PW can be effectively counterbalanced by N fertilization [45]. Though the presence of startup nutrients in growing substrate has been considered a favorable trait, precise nutrient management during cultivation has made it less important.

### 4.3. Phytotoxicity and Plant Cultivation

Germination index for all the SE treatments was dramatically lower than 50% indicating a high phytotoxicity [26]. Therefore, phytotoxicity could be a major factor limiting the application of PW as growing substrate. Phytotoxicity of PW may originate from the accumulation of phytotoxic chemicals, particularly phenol [46]. Increased phenol in SE-treated PW is possibly due to the degradation of lignin. Lignin is a polyphenolic polymer. The β-O-4 aryl ether bond, which is the most abundant linkage in lignin macromolecules, is prone to hydrolysis following SE, releasing free phenolic groups [47].

Torrefaction is a widely used thermal pretreatment of biomass mainly for solid fuel production [48]. This study indicate that torrefaction would effectively reduce phytotoxicity of PW. The gas and liquid products of torrefaction contain mainly monoaromatics, heterocyclic hydrocarbons, aldehyde, alcohol, and ketone [31], which may be derived from degradation of phytotoxins. Our preliminary data indicate that torrefaction at 300 °C for 30 min could further decrease the concentration of total phenol and total flavonoid in SE-treated PW by 61.6 and 9.6%, respectively, compared to that for 15 min (Figure S2). Such results suggest that more effective removal of phytotoxicity could be achieved by modifying torrefaction conditions.

As the pretreatment severity of SE increased, shoot biomass of bok choy gradually decreased possibly due to the increased phytotoxicity. However, subsequent torrefaction brought shoot biomass grown in SE-treated PW with high pretreatment severity to a level that was similar to that with low pretreatment severity. Such results also suggest that torrefaction is effective in phytotoxicity removal. In the absence of torrefaction, SE-treated PW may only be suitable for low peat substitution percentage such as 5 and 10%. However, PW received a combined treatment of SE and torrefaction may substitute peat for a higher percentage. 

Shoot biomass with replaced peat by treated pruning waste is still lower than 100% peat, particularly for those with 20 and 40% substitution. It is thus necessary to determine whether the yield loss could be compensated by the reduced input due to less peat usage before SE-treated PW can be used as a peat alternative. 

## 5. Conclusions

Evaluated SE conditions altered physiochemical properties of PW as soilless growing substrate, such as bulk density, porosity, pH, EC, chemical compositions, and acid soluble nutrients. Temperature and retention time had inconsistent impacts on such properties. Phytotoxicity is a major factor restricting the use of SE-treated PW as growing substrate. Higher SE pretreatment severity may cause higher phytotoxicity. Torrefaction could effectively reduce phytotoxicity and SE may facilitate the removal efficiency. Pruning waste may be used as peat alternative for up to 40% following certain SE and/or torrefaction treatments.

## Figures and Tables

**Table 1:** Physical properties of pruning waste as affected by main effects of temperature and retention time.

Treatment ^1^	BD ^2^	TP	WHC	AFP
Temperature				
°C	g cm^−3^	––––––––––% v/v––––––––––		
160	0.26 b	74.6 ab	65.8 b	8.8 ns
175	0.27 b	77.9 a	71.8 a	6.1
190	0.28 ab	75.9 ab	68.3 ab	7.6
205	0.29 ab	73.2 b	67.8 ab	5.4
220	0.31 a	71.9 b	66.6 ab	5.4
Retention time				
min	g cm^−3^	––––––––––% v/v––––––––––		
1	0.27 B	74.1 AB	67.2 AB	6.9 ns
3	0.28 AB	73.0 B	66.5 B	6.6
5	0.30 A	76.9 A	70.5 A	6.5

^1^ Abbreviations: BD = bulk density; TP = total porosity; WHC = water-holding capacity; AFP = air-filled porosity. ^2^ Means within each column followed by different lower-case or upper-case letters indicate significant differences among temperatures or retention times at α = 0.05. ns indicates no significant difference.

**Table 2:** Chemical properties of pruning waste as affected by temperature × retention time interaction.

Temperature ^1^	Time	pH ^2^	EC	CEC	Chemical Composition			Elemental Analysis			Acid Soluble Nutrient		
					Hemicellulose	Cellulose	Lignin	C	N	C/N	Na	Ca	P
°C	Min		mS cm^−1^	cmol_c_ kg^−1^	–––––––––mg g^−1^–––––––––			––––%––––			–––––mg kg^−1^–––––		
160	1	5.99 a	0.82 i	9.9 ab	222 a	381 b	313 a	45.1 d	0.33 ns	137 a	74 bc	408 b	575 ns
	3	5.79 b	0.93 h	11.4 a	220 a	360 b	273 b	45.2 d	0.42	109 ab	62 bc	459 ab	575
	5	5.58 c	1.12 fg	9.4 ab	200 b	397 ab	305 ab	45.4 d	0.47	98 b	111 a	576 ab	713
175	1	5.83 b	1.04 g	7.8 b	201 b	346 b	294 ab	45.4 d	0.45	102 b	102 ab	601 a	627
	3	5.69 bc	1.11 fg	6.0 b	204 b	416 a	282 ab	45.3 d	0.33	137 ab	80 b	497 ab	600
	5	5.35 d	1.05 g	8.8 ab	177 c	416 a	288 ab	46.4 c	0.34	139 a	48 c	392 b	548
190	1	5.63 c	1.16 f	11.3 ab	180 c	365 b	274 b	46.1 cd	0.42	110 ab	98 ab	571 ab	679
	3	5.36 d	1.43 d	10.4 ab	156 d	350 b	234 c	46.5 c	0.43	108 ab	83 ab	547 ab	714
	5	5.05 e	1.41 d	8.4 b	142 e	413 a	280 b	46.8 bc	0.40	118 ab	81 b	414 b	699
205	1	5.27 de	1.27 e	6.7 b	159 d	398 ab	263 bc	45.7 cd	0.38	120 ab	80 b	544 ab	688
	3	5.16 e	1.79 ab	6.9 b	95 g	388 ab	279 b	46.4 c	0.45	104 ab	103 ab	551 ab	713
	5	5.02 ef	1.73 b	11.1 ab	83 h	373 b	266 b	47.5 b	0.45	107 ab	95 ab	528 ab	750
220	1	5.05 e	1.35 de	11.1 ab	129 f	366 b	288 ab	46.6 c	0.39	120 ab	59 bc	422 b	624
	3	4.90 f	1.54 c	8.3 b	70 i	334 b	273 b	48.3 ab	0.36	134 ab	65 bc	465 ab	736
	5	4.77 f	1.83 a	7.3 b	74 hi	336 b	256 bc	48.8 a	0.48	102 b	88 ab	471 ab	784

^1^ Abbreviations: EC = electric conductivity; CEC = cation exchange capacity; C = total carbon; N = total nitrogen. ^2^ Means within each column followed by different lower-case letters indicate significant different differences at α = 0.05, and ns indicate no significant differences at α = 0.05.

**Table 3:** Concentration of total phenol and total alkaloid as affected by temperature × retention time interaction.

Temperature	Time	Alkaloid ^1^	Phenol
°C	Min	–––––––mg g^−1^–––––––	
160	1	2.0 i	11.1 e
	3	2.4 h	11.1 e
	5	4.4 e	12.4 de
175	1	2.6 gh	10.7 e
	3	5.7 b	14.6 de
	5	5.2 c	14.6 de
190	1	4.7 d	16.1 de
	3	2.3 h	20.9 cd
	5	3.9 f	24.3 cd
205	1	5.5 bc	18.4 d
	3	9.1 a	25.9 c
	5	4.7 d	38.0 b
220	1	4.9 d	23.6 cd
	3	4.6 de	42.3 b
	5	2.9 g	51.5 a

^1^ Means within each column followed by different lower-case letters indicate significant different differences at α = 0.05.

**Table 4:** Germination index (GI), concentration of total alkaloid, phenol, and flavonoid, pH, electrical conductivity (EC), total carbon (C), total nitrogen (N), and C/N ratio as affected by steam explosion×torrefaction interaction.

Steam Explosion	Torrefaction	GI ^1^	Alkaloid	Phenol	Flavonoid	pH	EC	C	N	C/N
		%	–––––––––––mg g^−1^–––––––––––				mS cm^−1^	–––––––%–––––––		
Untreated	No	4.3 c	1.0 e	10.2 d	18.0 c	6.58 a	0.85 cd	44.6 f	0.28 c	159 a
	Yes	23.0 b	0.8 f	14.9 cd	7.4 e	6.42 b	0.79 d	48.8 c	0.40 bc	122 b
160 °C + 3 min	No	16.2 b	2.4 b	11.1 d	22.7 b	5.79 c	0.93 cd	45.2 e	0.42 b	109 b
	Yes	46.2 a	1.9 c	15.1 cd	7.8 de	6.50 ab	0.83 cd	49.9 b	0.46 ab	108 b
190 °C + 3 min	No	6.5 c	2.3 b	20.9 c	26.5 ab	5.36 d	1.43 b	46.5 d	0.43 b	108 b
	Yes	54.7 a	2.9 a	18.3 c	9.1 de	6.47 ab	0.95 cd	50.2 b	0.43 b	118 b
220 °C + 5 min	No	0.7 c	2.9 a	51.5 a	29.2 a	4.77 e	1.83 a	48.8 c	0.48 ab	102 b
	Yes	52.1 a	1.6 d	28.3 b	11.8 d	6.42 b	0.98 c	53.1 a	0.57 a	95 b

^1^ Means within each column followed by different lower-case letters indicate significant differences at α = 0.05.

## Data Availability

Not applicable.

## Supplementary Materials

The following supporting information can be downloaded at: https://www.mdpi.com/article/10.3390/ijerph19095328/s1, Figure S1: Image (A) and schematic diagram (B) of the instant catapult steam explosion (ICSE) apparatus; Table S1: Temperature, pressure, retention time, and pretreatment severity (LogR_0_) of steam explosion treatments evaluated in this study; Figure S2: Concentration of total phenol and total flavonoid in pruning waste treated by steam explosion at 160 °C + 3 min with and without torrefaction. Data represent mean ± SE. Different lower-case and upper-case letters indicate significant differences among treatments for phenol and flavonoid, respectively, at α = 0.05.
