# Supplementary material for: Steam-Exploded Pruning Waste as Peat Substitute: Physiochemical Properties, Phytotoxicity and Their Implications for Plant Cultivation
Source: Int J Environ Res Public Health. 2022 Apr 27;19(9):5328. doi: 10.3390/ijerph19095328 (PMC9103252; doi:10.3390/ijerph19095328)
Supplement: Supplementary file 1 [file ijerph-19-05328-s001.zip › ijerph-1688148-supplementary.pdf]

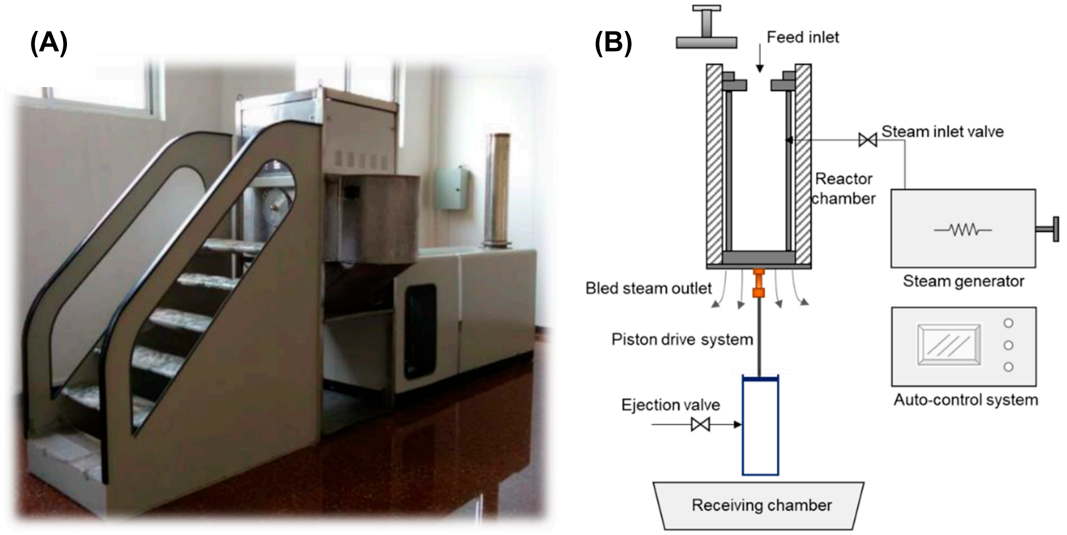

Figure S1 Image (A) and schematic diagram (B) of the instant catapult steam explosion (ICSE) apparatus

Table S1 Temperature, pressure, retention time, and pretreatment severity ($LogR_0$) of steam explosion treatments evaluated in this study

Temperature	Pressure	Time	$LogR_0$ [a]
°C	MPa	Min	
160	0.6	1	1.77
		3	2.24
		5	2.47
175	0.9	1	2.21
		3	2.69
		5	2.91
190	1.3	1	2.65
		3	3.13
		5	3.35
205	1.7	1	3.09
		3	3.57
		5	3.79
220	2.3	1	3.53
		3	4.01
		5	4.23

[a] Pretreatment severity ($LogR_0$) is defined as $\log R_0 = \log(t \times e^{\frac{T-100}{14.75}})$, where t is the pretreatment time in minutes and T is the pretreatment temperature in degrees Celsius.

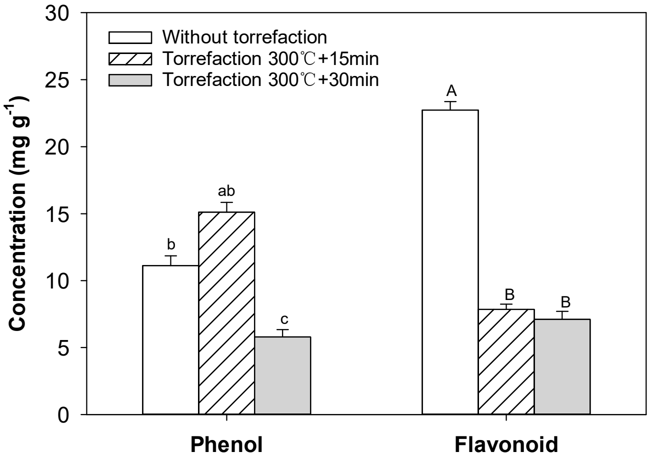

Figure S2 Concentration total phenol and total flavonoid in pruning waste treated by steam explosion at 160°C+3 min with and without torrefaction. Data represents mean ± SE. Different lower-case and upper-case letters indicate significant differences among treatments for phenol and flavonoid, respectively, at α=0.05.
